# MXene-Based Electrodes for Flexible Supercapacitors: From Material Synthesis to Device Integration

**DOI:** 10.3390/ma19122618

**Published:** 2026-06-17

**Authors:** Wenlong Luo, Hongyu Zhao, Qingrong Li, Cai Liang, Jing Sun, Xinyan Zhang, Yingping Pang, Yanpeng Mao, Zhanlong Song, Ziliang Wang

**Affiliations:** 1 College of Physics and Electrical Engineering, Kashi University, Kashi City 844000, China; hongyu_zhao@ksu.edu.cn (H.Z.); qrl1783488390@163.com (Q.L.); 2 National Engineering Laboratory for Reducing Emissions from Coal Combustion, Engineering Research Center of Environmental Thermal Technology of Ministry of Education, Shandong Key Laboratory of Green Thermal Power and Carbon Reduction, Shandong University, Jinan 250061, China; 3 Key Laboratory of Energy Thermal Conversion and Control of Ministry of Education, School of Energy and Environment, Southeast University, Nanjing 211102, China

**Keywords:** MXene, electrodes, synthetic strategy, composites, flexible supercapacitors

## Abstract

With the rapid advancement of portable wearable electronics, flexible supercapacitors have ushered in new development opportunities. In recent years, MXene and its composites have demonstrated potential as advanced supercapacitor electrode materials due to their outstanding theoretical capacitance, specific surface area, conductivity, hydrophilicity, and mechanical flexibility. This review traces the development of MXene and summarizes common synthesis strategies, with a focus on the effects of different preparation methods on its structure and properties. Departing from previously reported work, this review draws from the practical requirements of flexible supercapacitors to conduct an in-depth analysis of the key factors influencing the charge storage, rate capability, cycling life, and mechanical flexibility of the devices. It summarizes common design strategies for MXene composites currently used to enhance device performance. Additionally, this study analyzes key challenges facing MXene-based electrode materials, including issues such as self-stacking of layers, insufficient oxidation stability, limited energy density, and structural degradation under complex deformation conditions. Mitigation strategies are summarized, including optimizing synthesis methods and constructing composite systems integrating carbon materials, conducting polymers, and transition metal compounds. Finally, future research directions for MXene in flexible energy storage are explored, emphasizing the need to achieve a balance between performance and manufacturability through synergistic regulation at structural design, interfacial engineering, and device levels. This review aims to provide theoretical guidance for the development of practical MXene-based wearable energy storage devices.

## 1. Introduction

Advances in emerging technologies such as wearable electronics, smart sensors, and soft robotics have driven higher demands for the characteristics of portable energy storage devices [1,2]. Among numerous promising storage solutions, flexible supercapacitors (SCs) are recognized as a key technology for advancing flexible portable energy storage equipment [3,4,5]. This is due to their advantages, including fast charge/discharge rates, high power density, outstanding mechanical flexibility, and a long life cycle. Recently, the focus of research on flexible SCs has gradually shifted from the sole pursuit of high specific capacitance and high energy density to a balance between electrochemical performance and device practicality. Among these, flexible SCs made from MXene have attracted widespread attention. This is due to their potential to achieve high energy density, excellent mechanical flexibility, long-term cycling stability, and scalability.

SCs primarily consist of three components: electrode materials, an electrolyte, and a separator. Among these, electrode materials are the core factor influencing SC performance [6]. Among the various electrode materials, carbon materials (such as activated carbon, graphene, and carbon nanotubes), conductive polymers (such as polyaniline, polypyrrole, and PEDOT), transition metal compounds (such as metal oxides, metal hydroxides, and metal sulfides), and MXenes are widely used in flexible SCs [7]. Among these, carbon materials exhibit excellent electrical conductivity, good chemical stability, and an outstanding cycling life. However, their energy storage process relies on the electrochemical double-layer mechanism (rapid physical desorption at the electrode surface), resulting in relatively low specific capacitance and energy density [8,9]. As such, they struggle to meet the demands of high-performance flexible energy storage devices. Conductive polymers possess high theoretical specific capacitance and good flexibility and can deliver high energy density through a pseudocapacitive mechanism (rapid and reversible Faradaic redox reactions). However, they are prone to volume expansion and contraction during repeated charging and discharging cycles, leading to structural degradation and reduced cycling stability [10,11,12]. Transition metal compounds possess abundant redox-active sites and high theoretical capacities, which can significantly enhance the energy density of SCs. However, they generally suffer from poor conductivity, slow ion diffusion kinetics, and insufficient mechanical flexibility, which limit their further applications in flexible energy storage [12,13,14].

MXene, as an emerging star among 2D materials, demonstrates tremendous application potential in flexible SCs due to its unique layered structure, abundant surface functional groups, and outstanding conductivity [15,16,17]. Compared to traditional carbon materials, MXene not only exhibits metal-level electrical conductivity but also participates in pseudocapacitive reactions through surface functional groups, thereby achieving higher charge storage capacity. Compared to conductive polymers, MXene offers a superior life cycle and structural stability. Unlike transition metal compounds, MXene combines high electrical conductivity with rapid ion transport, which facilitates excellent rate performance [18,19]. Beyond this, MXene’s layered structure, tunable interlayer spacing, abundant surface functional groups, and good hydrophilicity provide favorable conditions for ion diffusion and interfacial reactions [19,20]. However, MXene still faces challenges, such as interlayer re-stacking, susceptibility to oxidation in water or air, and challenges related to large-scale production [21,22].

To better serve SCs, researchers have undertaken numerous attempts within the MXene family. Venkateshalu et al. [23] prepared an accordion-like Ti_3_C_2_T_x_ MXene via conventional HF etching. In a standard three-electrode system using 6 M of KOH as the electrolyte, the multilayer Ti_3_C_2_T_x_ MXene electrode exhibited a gravimetric capacitance of 197 F g^−1^ at 1 A g^−1^. Chang et al. [24] utilized an in situ HF (HCl + LiF) etching method to prepare MXene dispersions, subsequently obtained MXene films via vacuum-assisted filtration, and combined them with VHB tape to form MXene/elastomer electrodes. In a 1 M Li_2_SO_4_ electrolyte, this electrode delivered a gravimetric capacitance of 118 F g^−1^ at 1 A g^−1^. Although its energy storage efficiency requires improvement, the electrode demonstrated excellent stretch resistance (96% retention after 1000 cycles at 50% strain). Wang et al. [25] synthesized Ti_3_C_2_T_x_ MXene nanosheets with fewer than five molecular layers by combining the minimal stratification method with a freeze-assisted exfoliation process. Electrochemical testing revealed that the freeze-exfoliated MXene nanosheets exhibited a mass-specific capacitance of 255 F g^−1^ under conditions of 1 M of H_2_SO_4_ and 2 mV s^−1^. Even at 200 mV s^−1^, they delivered a high mass-specific capacitance of 150 F g^−1^ (six times that of unfrozen-exfoliated MXene), demonstrating the excellent rate capability of few-layer MXene. Studies have shown that strategies such as surface modification, material intercalation, and heterostructure construction can effectively enhance the specific capacitance and cycling stability of MXene-based electrodes [26,27,28]. Therefore, optimizing electrode structures to improve ion transport efficiency, extend the life cycle, enhance energy density, and maintain mechanical flexibility represents a core challenge in designing MXene-besed flexible SCs to elevate device performance.

**Figure 1:**
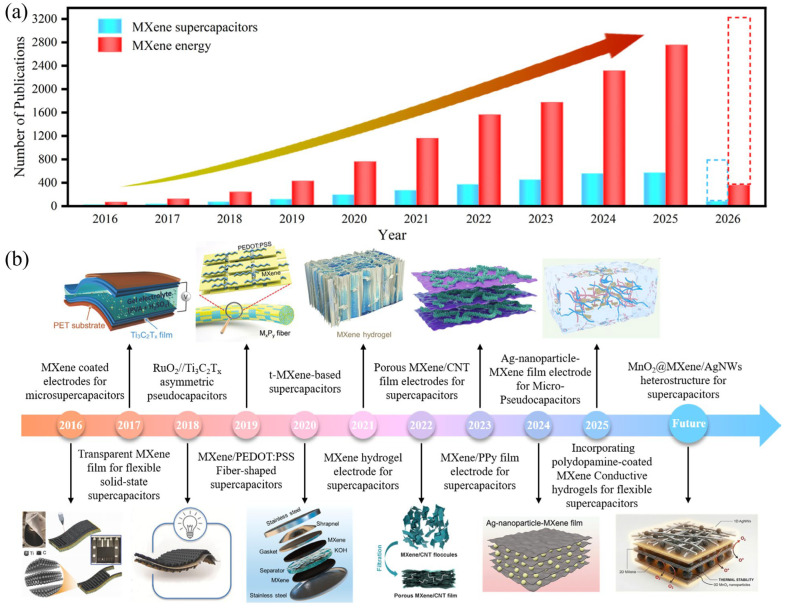
(**a**) The annual publication trend of MXene applications in supercapacitors and energy fields since 2016. (Data source: Web of Science; Keywords: MXene and Supercapacitors, MXene and energy). (**b**) The development of MXene-based materials for flexible supercapacitors. Reproduced with permission [29]. Copyright 2016 WILEY-VCH GmbH. Reproduced with permission [30]. Copyright 2017 WILEY-VCH GmbH. Reproduced with permission [31]. Copyright 2018 WILEY-VCH GmbH. Reproduced with permission [32]. Copyright 2019 WILEY-VCH GmbH. Reproduced with permission [33]. Copyright 2020 American Chemical Society. Reproduced with permission [34]. Copyright 2021 The Authors. Reproduced with permission [35]. Copyright 2022 American Chemical Society. Reproduced with permission [36]. Copyright 2023 Elsevier B.V. Reproduced with permission [37]. Copyright © 2024 Wiley-VCH GmbH. Reproduced with permission [38]. Copyright 2025 American Chemical Society. Reproduced with permission [39]. Copyright 2026 The Authors.

Since 2016, the number of published articles on MXene applications in SCs and energy storage has shown a stepwise increase, as depicted in Figure 1a. Lei et al. [40] reviewed the diverse properties of MXene and its applications across various energy storage devices, identifying several future challenges for MXene in energy storage. Fang et al. [41] summarized design strategies and performance optimization for fiber-based SCs. Jiang et al. [42] discussed recent research advances on MXene in solid-state SCs, introducing emerging electrode structures for solid-state SCs. Although many studies have reviewed progress in MXene research in the fields of SCs and energy storage, their scope is either too broad or lacks timeliness. Therefore, to systematically review the latest research progress on MXene-based flexible SCs, this paper first reviews the development history of MXene (Figure 1b) [29,30,31,32,33,34,35,36,37,38,39], focusing on how different preparation methods affect MXene structure and performance. Subsequently, based on research findings from the past five years, this paper systematically summarizes the latest research progress on MXene and its composites in flexible SCs. A comparison is made between different composite strategies and electrode structure designs and their effects on the electrochemical performance of the devices. Unlike existing research, this review focuses on the application characteristics of MXene in flexible SCs, with particular emphasis on the roles played by different structural designs and composite systems in enhancing charge storage, rate capability, cycling life, and mechanical flexibility. Furthermore, this review highlights the major challenges facing MXene in flexible SC applications and summarizes corresponding mitigation strategies. Finally, a rational outlook on the future development of MXene-based flexible SCs is presented. This paper aims to review the application of MXene in flexible SCs from the perspectives of MXene synthesis and composite structure design to provide theoretical guidance for the development of high-performance wearable energy storage systems.

## 2. Structure and Composition of MXene

**Figure 2:**
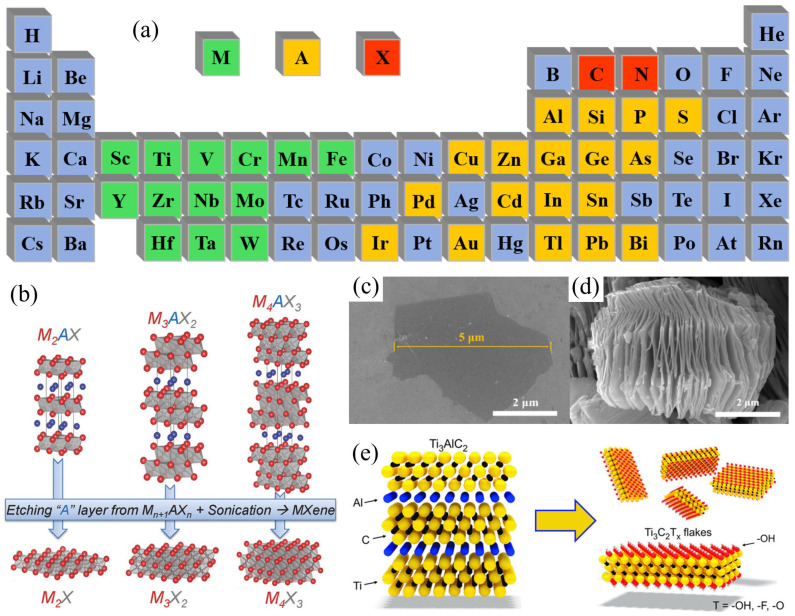
(**a**) The positions of each component within MAX phase. Green indicates elements in Layer M of the MAX phase; orange indicates elements in Layer A of the MAX phase; red indicates C/N elements in the MAX phase; and blue indicates other elements not involved in the composition of the MAX phase. (**b**) Crystal structure changes during MAX phase etching process. Reproduced with permission [46]. Copyright 2013 WILEY-VCH GmbH. SEM images of single-layer MXene (**c**) and Multilayer MXene (**d**). Reproduced with permission [48]. Copyright 2021 Elsevier Ltd. (**e**) Structural diagrams of Ti_3_AlC_2_ and Ti_3_C_2_T_x_. Recomposed with permission [49]. Copyright 2016 WILEY-VCH GmbH.

MXene has emerged as a rising star in the family of two-dimensional materials in recent years. It represents an early-stage transition metal carbide, nitride, or carbonitride, typically obtained by selectively removing the A layer from the precursor MAX phase [43,44]. Figure 2a illustrates the distribution of constituent elements of the MAX phase within the periodic table of elements. The general molecular formula for the MAX phase can be expressed as M_n+1_AX_n_ (n = 1–3), where M represents transition metals such as Ti, V, Zr, Nb, or Mo; A mainly denotes elements from Group III_A_ or IV_A_, such as Al or Si; and X signifies C and/or N elements [45]. From the molecular structure shown in Figure 2b, the MAX phase exhibits a symmetrical hexagonal arrangement where M layers and A layers are nested. Within closely adjacent M layers, X occupies the central site of the octahedron [46,47]. In the MAX phase, M-X bonds exhibit covalent/metallic/ionic characteristics, while M-A bonds display typical metallic properties. Compared to the covalently bonded M-X bonds, the interlayer M-A bonds possess greater chemical reactivity. Consequently, MXene can be selectively obtained by etching away the A layer from MAX phase precursors. After etching, MXene reassembles from M-X bulk into a two-dimensional layered structure with graphene-like properties. Depending on the etching condition of layer A, it exhibits either an accordion-like multilayer structure or a flat-surfaced sheet (Figure 2c,d) [48]. The chemical formula for MXene is typically represented as M_n+1_X_n_T_x_ (n = 1–3), where M and X denote transition metals and C/N elements, respectively, and T_x_ indicates the terminal functional groups (-O, -OH, -F, -Cl, etc.) present on the MXene surface along with their quantities (Figure 2e) [49]. The formation of these functional groups is influenced by MXene preparation methods, and their variations affect the conductivity and surface hydrophilicity of MXene [50]. To date, over 100 single-metal or bimetallic MAX phases have been identified, but only more than 30 MXenes—including Ti_3_C_2_T_x_, Ti_2_CT_x_, Ti_3_CNT_x_, Mo_2_CT_x_, and Nb_4_C_3_T_x_—have been synthesized [51,52,53,54,55]. Notably, among these successfully synthesized MXenes, Ti_3_C_2_T_x_—the first MXene synthesized—has seen the most extensive applications [56].

## 3. Synthetic Strategies for MXene

Currently, reported MXene preparation methods are primarily categorized into top–down and bottom–up approaches, depending on whether their two-dimensional structure originates from MAX precursors or is directly constructed from molecules or atoms [57,58]. It is worth noting that differences in synthesis techniques directly impact structural integrity and surface chemistry, thereby determining the final electrochemical performance.

### 3.1. Top–Down

The top–down synthesis strategy is achieved through selective etching of the A layer in MAX phase precursors. This approach exploits the bond energy difference between M–X and M–A bonds to remove A elements while preserving the M_n+1_X_n_ layered structure. Top–down strategies primarily include HF etching [59,60], fluoride salt etching [61,62], molten salt etching [63,64], and electrochemical etching [65,66,67].

#### 3.1.1. HF Etching

HF etching is currently the most established and widely used method for preparing MXene [68]. Its core mechanism involves the selective breaking of M-A bonds in the MAX phase by HF, thereby stripping away the A-layer atoms and preserving the two-dimensional M-X framework structure. However, HF etching of the MXA phase is not a simple process of A-atom delamination. It is accompanied by processes such as defect formation, surface termination, and interlayer restructuring, all of which collectively determine the final electrochemical performance of MXene.

**Figure 3:**
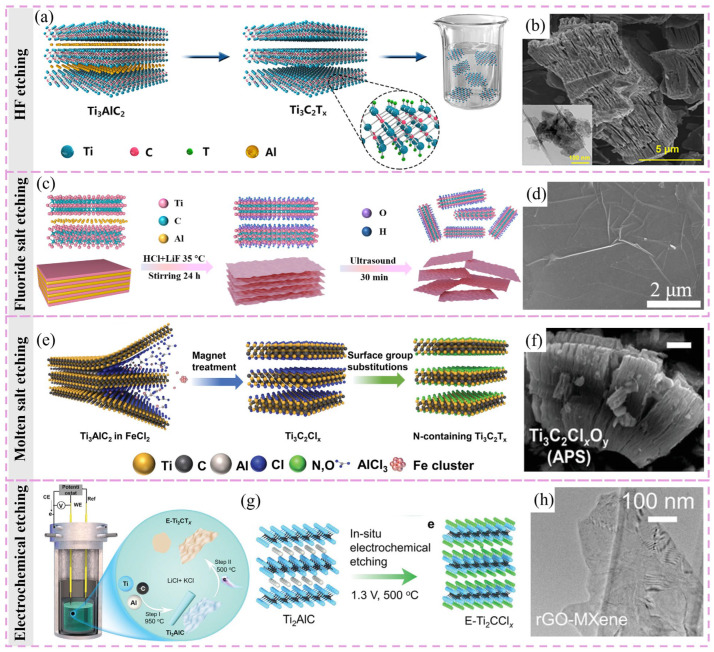
(**a**) HF etching method for MXene synthesis. (**b**) SEM image of MXene by HF etching method. Reproduced with permission [81]. Copyright 2017 Elsevier B.V. Process flow (**c**) and SEM image (**d**) of MXene synthesis via HCl + LiF etching method. Reproduced with permission [82]. Copyright 2023 Elsevier Inc. Process flow (**e**) and SEM image (**f**) of MXene synthesis via molten salt etching method (scale bar in the image is 2 µm). Reproduced with permission [83]. Copyright 2020 The Authors. Process flow (**g**) and SEM image (**h**) of MXene synthesis by electrochemical etching method. Reproduced with permission [84]. Copyright 2022 The Authors.

Taking the preparation of Ti_3_C_2_T_x_ from the most typical Ti_3_AlC_2_ as an example, we analyze the MXene synthesis process (Figure 3a,b). The etching of Ti_3_AlC_2_ involves multiple reactions, with the corresponding chemical equations as follows:

(1) $${\mathrm{Ti}}_{3}{\mathrm{AlC}}_{2}+3\mathrm{HF}={\mathrm{AlF}}_{3}+{\mathrm{Ti}}_{3}C_{2}+\frac{3}{2}H_{2}$$

(2) $${\mathrm{Ti}}_{3}C_{2}+2H_{2}O={\mathrm{Ti}}_{3}C_{2}{\left(\mathrm{OH}\right)}_{2}+H_{2}$$

(3) $${\mathrm{Ti}}_{3}C_{2}+2\mathrm{HF}={\mathrm{Ti}}_{3}C_{2}F_{2}+H_{2}$$

Among them, Equation (1) represents the key step for obtaining layered Ti_3_C_2_T_x_ MXene, while Equations (2) and (3) determine the terminal groups (-O, -OH, and -F) on the MXene surface [69]. Studies have shown that HF concentration, reaction time, and temperature are important factors affecting the structure and properties of MXene, with HF concentration being the key factor determining MXene structure. Low-concentration HF (≤10 wt.%) can effectively suppress lattice defects and reduce the content of -F functional groups, but this also makes it difficult to etching the Al completely [70,71]. Conversely, while high-concentration HF (≥40 wt.%) can shorten the etching time, it can cause irreversible defects, such as Ti/C vacancies or edge damage [69]. Furthermore, high-concentration HF leads to an increase in the content of -F functional groups on the MXene surface, thereby impairing charge transport. In contrast, 20–30 wt.% HF provides a better balance between etching efficiency, structural integrity, and terminal functional groups, making it easier to obtain MXene materials with excellent electrochemical performance [72]. Table 1 compares the effects of different HF concentrations on the structure and electrochemical performance of Ti_3_C_2_T_x_ MXene.

Notably, while the HF etching method offers advantages such as a mature process, low cost, high synthesis efficiency, and broad applicability, concentrated HF is inherently corrosive and highly toxic, which severely limits the large-scale production of MXene by this method. Furthermore, MXene obtained via HF etching often exhibits smaller interlayer spacing and a high density of -F functional groups. This results in reduced MXene conductivity and impedes electrolyte ion diffusion, thereby compromising the electrochemical performance of MXene.

#### 3.1.2. Fluoride Salt Etching

For the large-scale preparation of MXene, the fluoride salt etching method is considered the most representative improvement strategy following direct HF etching. The core strategy involves the in situ synthesis of HF using fluoride salts such as LiF [31], NH_4_F [73], NaF [74], FeF_3_ [75], or NH_4_HF_2_ [76] in a strong acid environment, thereby enabling selective etching of the MAX phase. Among these, the concentrated HCl/LiF system is currently the most widely used in situ HF etchant for MXene synthesis [77], with the following in situ reaction equation:

(4) HCl+LiF=HF+LiCl

As shown in Figure 3c,d, the insertion of Li^+^ between layers during the etching process effectively increases the interlayer spacing and facilitates subsequent exfoliation, resulting in low-defect few-layer/single-layer MXene nanosheets [78]. Remarkably, these low-defect few-layer/single-layer MXene nanosheets exhibit superior electrochemical performance compared to multilayer MXene. Compared to direct HF etching, the method of generating HF in situ using fluoride salts in combination with strong acids offers enhanced safety. Furthermore, metal cation intercalation effectively weakens van der Waals forces between adjacent nanosheets, promoting MXene exfoliation. This process increases interlayer spacing or yields few-layer/single-layer MXene nanosheets, thereby enhancing ion transport capabilities during electrochemical reactions [79,80].

Although the fluoride salt etching method has improved the safety and exfoliation efficiency of MXene preparation, it has not fully resolved the inherent issues of the HF system; the etching process still inevitably introduces many -F functional groups. Furthermore, the cations introduced by different fluoride salts can significantly alter the defect structure and interlayer chemical environment of MXene. For example, the LiF/HCl system tends to yield larger interlayer spacings and better exfoliation properties, whereas the FeF_3_/HCl system may introduce metal ion residues or even form localized heterogeneous phases, leading to a more complex material composition [75]. Studies have shown that Ti_3_C_2_T_x_ materials prepared using different fluoride salt systems exhibit significant differences in terminal group composition, oxidative stability, flake size, and defect concentration. These differences ultimately manifest in their electrochemical performance. Table 2 compares the advantages and limitations of different fluoride salt systems.

#### 3.1.3. Molten Salt Etching

Distinct from traditional HF etching and fluoride salt etching methods, the molten salt etching method utilizes a high-temperature molten Lewis acid salt to undergo a redox reaction with the MAX phase, selectively removing atoms from the A layer to obtain layered MXene [85]. The advantage of this method lies in its ability to minimize the introduction of -F functional groups and to control the terminal functional groups of MXene, thereby providing new degrees of freedom for MXene’s surface chemistry. As shown in Figure 3e,f, studies have revealed that MXene surfaces etched by molten salts exhibit a greater tendency to form -Cl, -O, or near-exposed metal sites [83]. Theoretically, this enhances electron transport capabilities and pseudocapacitive reactivity, offering potential advantages for supercapacitor and battery electrodes. Furthermore, this method overcomes the etching limitations inherent to certain traditional MAX phases, thereby expanding the compositional possibilities for MXene.

However, molten salt etching typically requires higher reaction temperatures, placing greater demands on equipment and energy consumption. MXenes prepared via this method lack -OH terminal groups, resulting in hydrophobicity that is undesirable for use as electrode materials. Furthermore, MXene typically exhibits a multilayer structure when prepared using a molten salt system, with strong interlayer bonding that makes subsequent exfoliation significantly more difficult than in fluoride salt systems [86]. Although improved strategies, such as LiF-assisted molten salt etching, have enhanced the stripability of MXene nanosheets, the overall process remains complex and costly [87]. More importantly, metal ions left behind during the Lewis acid etching process may become embedded between layers or form secondary phases, leading to a more complex material composition and thereby affecting its electrochemical properties [88]. Overall, molten salt etching remains an immature technique, highly susceptible to process parameters such as molten salt properties and temperature. Its applicability and the structural stability of the resulting products still lack systematic investigation.

#### 3.1.4. Electrochemical Etching

Electrochemical etching is a strategy for preparing MXene driven by an applied electric field. This method typically employs the MAX phase as an electrode, where layer A elements are removed through anodic dissolution or selective etching in a specific electrolyte [89]. Referring to Figure 3g,h, Liu et al. [84] successfully achieved the transformation of the Ti_2_AlC MAX precursor into Ti_2_CT_x_ MXene through electrochemical etching in a LiCl-KCl solution. Subsequently, further treatment of Ti_2_CT_x_ MXene with ammonium persulfate introduced -O functional groups that synergistically interact with -Cl groups, thereby enhancing redox reactions of MXene in non-aqueous electrolytes and improving its charge storage capacity. Compared to chemical etching, the prominent advantage of electrochemical etching lies in its ability to precisely control the etching depth of the MAX phase, the interlayer structure and the surface termination groups for MXene, by adjusting the potential, current, and time during the etching process [89]. Electrochemical etching makes it easier to obtain large-sized MXene flakes with low defect rates. Furthermore, this method involves mild reaction conditions, offering a viable route for the green synthesis of MXene.

Electrochemical etching is one of the most promising green strategies for MXene synthesis, but it remains in the exploratory stage, with improvements needed in process maturity and universality. Furthermore, although electrochemical etching can effectively reduce the content of -F groups, it suffers from low etching efficiency, long reaction times, limited applicability to MAX systems, and the relative sensitivity of MAX to electrochemical conditions [90]. The electrochemical etching process requires strict control of process parameters such as electrolyte composition and applied voltage; inadequate control can easily lead to defects or incomplete etching. To provide a clearer understanding of the application of electrochemical etching in MXene preparation, Table 3 summarizes the advantages and limitations of different electrochemical etching systems for MXene synthesis.

All in all, HF etching and fluoride salt etching remain the mainstream strategies for MXene synthesis. Melt salt etching and electrochemical etching represent significant developments in advancing top–down MXene preparation from “achievable” to “controllable.” These methods show promises in reducing -F functional group content and optimizing surface chemistry, though their maturity and universality still require further refinement.

### 3.2. Bottom–Up

**Figure 4:**
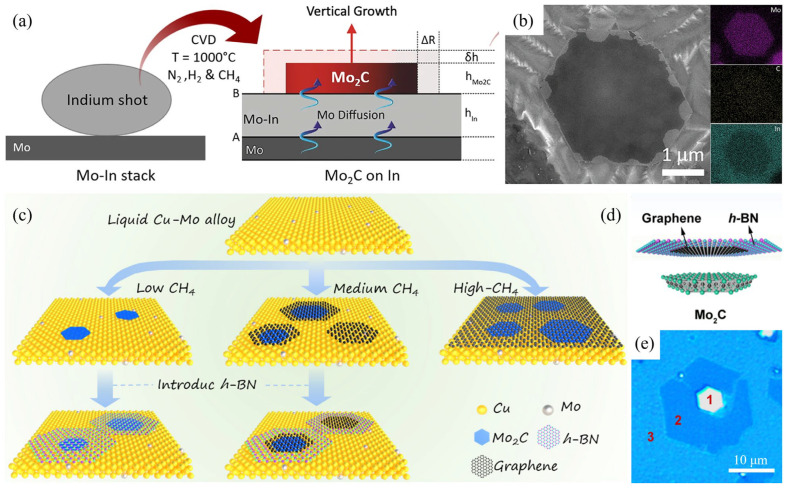
(**a**) Process flow for synthesizing Mo_2_C MXene by chemical vapor deposition on In bulk. (**b**) SEM image and EDS elemental spectrums of Mo_2_C MXene. Reproduced with permission [98]. Copyright 2021 The Authors. (**c**) Process flow for preparing Mo_2_C MXene via chemical vapor deposition on Cu foil at different CH_4_ concentrations. (**d**) Structural schematic diagram and (**e**) SEM image of graphene/h-BN/Mo_2_C MXene. The numbers 1, 2, and 3 in the figure represent graphene/Mo_2_C staked block, monolayer graphene, and monolayer h-BN, respectively. Reproduced with permission [99]. Copyright 2025 Wiley-VCH GmbH.

Differing from top–down synthesis strategies, bottom–up synthesis techniques such as chemical vapor deposition (CVD) [91,92], the template method [93,94] and pulsed laser deposition (PLD) [95] directly construct the MXene framework from atomic or molecular units rather than etching MAX phase precursors [96]. Among these, CVD synthesis of the Mo_2_C MXene is a representative case of bottom–up MXene preparation [97]. Caylan and his colleagues [98] successfully converted CH_4_ and metallic Mo into large-area hexagonal Mo_2_C MXene films on a metallic In surface via CVD (Figure 4a,b). In this method, metallic In was first melted at high temperatures to cover the metallic Mo and form a Mo-In alloy. Subsequently, C atoms generated from CH_4_ cracking combined with diffusing Mo atoms at the Mo-In alloy interface to form a thin Mo_2_C layer. The study revealed that the thickness of Mo_2_C nanosheets inversely correlates with the thickness of the In layer. This phenomenon is attributed to the high concentration of molten In atoms hindering Mo atom diffusion, thereby limiting the vertical growth of Mo_2_C. Li et al. [99] reported a strategy for selectively synthesizing Mo_2_C MXene using CH_4_ as a carbon source by controlling the CH_4_ concentration during the CVD process (Figure 4c). Using copper foil as a template, this method enables structural and orientation modifications by adjusting the CH_4_ concentration, successfully yielding various vertical/horizontal heterostructures combining graphene, hexagonal boron nitride (h-BN), and Mo_2_C (Figure 4d,e). Additionally, Wang et al. [100] investigated the process parameters for synthesizing two-dimensional Mo_2_C nanosheets via CVD using CO and MoO_3_ as precursors. The study revealed that maintaining the reduction temperature between 950 and 1050 °C, with CO and Ar flow rates of 900 mL min^−1^ and 100 mL min^−1^, respectively, facilitates the formation of finely structured two-dimensional Mo_2_C sheets.

Bottom–up strategies enable precise control over the framework structure and chemical composition of MXene at the atomic level, yielding customized MXene with finer structures and enhanced stability. This approach opens new avenues for preparing single-layer or few-layer MXene. However, these techniques generally require expensive equipment and demanding reaction conditions, which undoubtedly increase energy consumption and production costs. Furthermore, the subsequent transfer and large-scale production of MXene, whether obtained via CVD or ALD, are currently not feasible.

## 4. Applications of MXene and Its Composites in Flexible Supercapacitors

Recently, the rapid advancement of portable flexible electronic devices has placed heightened demands on flexible SCs that combine high power density with outstanding stability [101]. Among numerous candidate materials, MXene has emerged as a critical system for a flexible SC design due to its high electrical conductivity, abundant surface functional groups, and controllable interlayer structure. However, pristine MXene materials suffer from issues such as interlayer self-stacking and mechanical flexibility instability. Consequently, structural regulation of MXene electrode materials and composite material construction have emerged as research hotspots. These approaches include building three-dimensional MXene frameworks [102,103], introducing carbon materials or conducting polymers to form synergistic networks [104,105,106,107], and designing heterojunctions to enhance electrochemical activity [108,109]. Based on this, this section discusses structural designs and representative examples of MXene and its composites in flexible SCs over the past five years, aiming to guide the advancement of flexible SCs and the application of MXene.

### 4.1. MXene Electrodes

**Figure 5:**
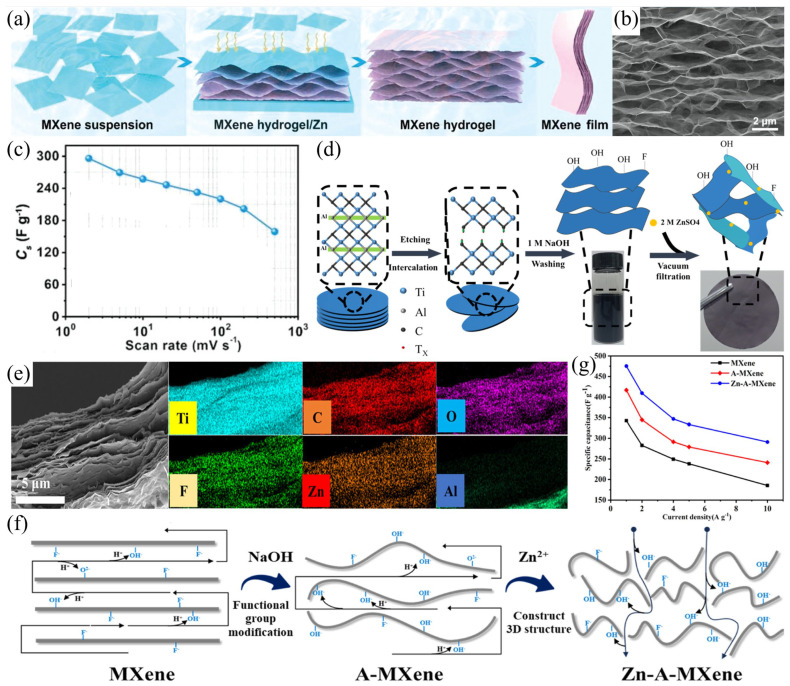
Schematic diagram of preparation process (**a**) and cross-section SEM image (**b**) for MXene film. (**c**) Relationship curve between scanning rate and the specific capacitance of the MXene film-2. Reproduced with permission [110]. Copyright 2021 Wiley-VCH GmbH. (**d**) Schematic diagram of synthetic pathway for Zn-A-MXene film. (**e**) SEM image and EDS elemental spectrums of Zn-A-MXene film. Schematic diagram of ion transport (**f**) and relationship curves between current density and the specific capacitance (**g**) of the different MXene films. Reproduced with permission [111]. Copyright 2023 American Chemical Society.

In flexible SCs, MXene electrodes serve multiple roles as conductive frameworks, active energy storage components, and structural regulation platforms. However, as electrode thickness and surface loading increase, their 2D layered structure is prone to self-stacking, which hinders ion diffusion and makes it difficult to achieve both high energy density and high power density. Therefore, how to construct efficient ion transport pathways while maintaining excellent conductivity has become one of the key challenges in MXene electrode design. To address this issue, Zhao et al. [110] prepared MXene films using a Zn substrate-assisted freeze-drying method and optimized their microstructure by adjusting the drying conditions (Figure 5a,b). The MXene film-2 exhibited a specific capacitance of 296 F g^−1^ (2 mV s^−1^) and a rate capability of 160 F g^−1^ (500 mV s^−1^) in 1 M of H_2_SO_4_ (Figure 5c), indicating that freeze-drying can effectively alleviate layer re-stacking and improve ion transport. However, the improvement in performance was relatively limited, indicating that relying solely on the regulation of physical pore structures cannot fundamentally resolve the issue of interlayer mass transfer limitations.

**Figure 6:**
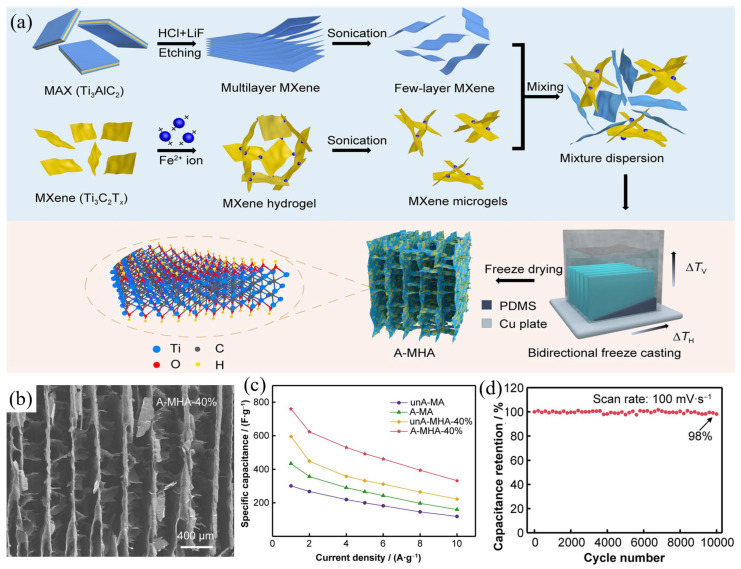
Schematic diagram of synthesis process (**a**) and SEM image (**b**) of A-MHA. (**c**) Relationship curves between current density and the specific capacitance of the different MXene aerogels. (**d**) Stability test of the A-MHA//CNT sponge ASC. Reproduced with permission [112]. Copyright 2023 Youke Publishing Co., Ltd.

In contrast, Xiao et al. [111] reduced the -F functional groups on the MXene surface through NaOH alkali washing and utilized Zn^2+^ electrostatic self-assembly to construct a three-dimensional cross-linked network (Figure 5d–f). The resulting Zn-A-MXene achieved a specific capacitance of 465.1 F g^−1^ at 1 A g^−1^, significantly outperforming both pure MXene and alkali-treated MXene (Figure 5g). Additionally, a flexible symmetric supercapacitor (SSC) assembled from Zn-A-MXene achieved an energy density of 9.55 Wh kg^−1^. However, the device retained only 81.25% of its initial capacitance after 5000 charge–discharge cycles, indicating that the metal–ion cross-linked structure suffers from stability issues during long-term cycling. Unlike previously reported work, Li et al. [112] prepared a bidirectionally oriented MXene composite aerogel (A-MHA) via bidirectional freeze-casting (Figure 6a,b). Benefiting from its unique structural design, A-MHA-40% exhibited a high specific capacitance of 760 F g^−1^ (1 A g^−1^) in 1 M of H_2_SO_4_, which is 2.5 times higher than that of MXene electrodes without a bidirectional alignment (Figure 6c). Compared to simply increasing porosity, designing an ordered ion transport network is more effective for enhancing the charge storage capacity of thick electrodes. However, although the asymmetric supercapacitors (ASCs) assembled from these electrodes exhibit excellent cycling stability (98% retention after 10,000 cycles) (Figure 6d), their energy density is only 3.4 Wh kg^−1^. This indicates that high specific capacitance does not necessarily translate into high device energy density and that a significant gap remains between electrode performance and device performance.

Overall, current MXene electrode designs primarily focus on establishing ion diffusion pathways, thereby effectively improving ion transport kinetics. However, most studies are still based on electrodes with low loading, and the issue of rapid mass transfer under high-loading conditions has not yet been fundamentally resolved. Meanwhile, some structural tuning strategies, while increasing specific capacitance, may reduce the device’s energy density and cycling stability. Therefore, future research should focus more on ion transport mechanisms and structural stability under high-loading conditions. By constructing hierarchical structures that combine high conductivity, ordered mass transfer channels, and a stable framework, it is possible to achieve a synergistic improvement in energy density, power density, and life cycle.

### 4.2. MXene/Carbon Materials’ Composite Electrodes

Carbon materials typically possess a high specific surface area and a rich pore structure, enabling them to form a continuous conductive network with MXene, thereby effectively mitigating the self-stacking issue of MXene nanosheets [113,114,115]. At the same time, the porous carbon scaffold promotes electrolyte wetting and ion diffusion while providing structural support during bending or stretching, thereby enhancing the mechanical stability of the electrode [116]. Consequently, MXene/carbon composite electrodes are considered one of the most representative strategies for improving the overall performance of flexible SCs. However, existing studies generally rely on complex multi-level structural designs to achieve performance improvements, and the structure–property relationship between the energy storage mechanisms and structural advantages remains to be further clarified.

**Figure 7:**
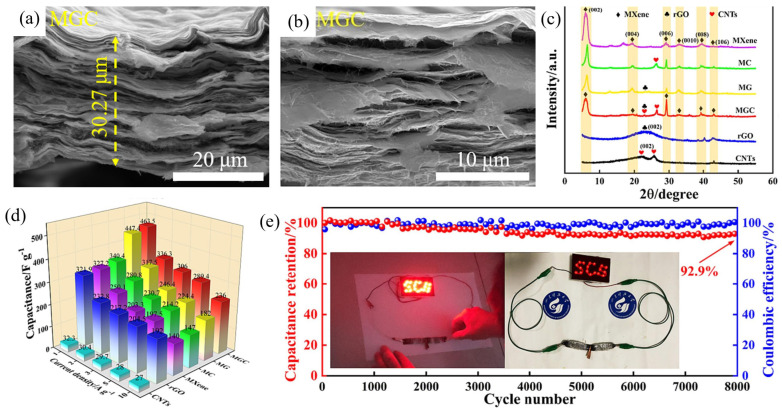
(**a**,**b**) A cross-section SEM image of MGC film. (**c**) XRD diffraction patterns of the different MXene films. (**d**) Histogram of change in specific capacitance with current density for different electrodes. (**e**) Stability test of the MGC//MnO_2_ ASC. Reproduced with permission [82]. Copyright 2023 Elsevier B.V.

Lyu et al. [117] fabricated a TSC aerogel composed of the Ti_3_C_2_ MXene, carboxylated single-walled carbon nanotubes (SWCNTs), and cellulose nanofibers (CNFs) using a supercritical drying method. The cross-linked fiber network formed by SWCNTs and CNFs effectively suppressed the stacking of MXene layers, enabling all TSC electrodes to exhibit a specific surface area exceeding 200 m^2^ g^−1^. The flexible SSC assembled by TSC3 aerogel electrodes demonstrated an areal capacitance of 746.68 mF cm^−2^ and a capacity retention of 91.23% (10,000 cycles). This work demonstrates that constructing a 3D network with high porosity can significantly enhance ionic accessibility and cycling stability. However, excessively high porosity typically accompanies a decrease in bulk density. This approach benefits high mass-to-capacity ratios, but it may compromise the device’s volumetric energy storage capacity. Addressing this issue is essential for the practical application of aerogel-based electrodes. Differing from 3D aerogels, Luo et al. [82] constructed a layered MXene/rGO/CNT (MGC) composite film using vacuum-assisted filtration (Figure 7a–c). Benefiting from the ionic transport channels formed by the synergistic intercalation of rGO and CNTs, the MGC electrode exhibited excellent energy storage performance under various testing conditions (Figure 7d). The MGC//MnO_2_ ASC assembled from this composite achieved an operating voltage window of 1.7 V and an energy density of 33.95 Wh kg^−1^, while maintaining good cycling stability and mechanical flexibility (Figure 7e). Compared to simply increasing porosity, this work demonstrates that a well-designed interlayer structure can simultaneously achieve both structural density and efficient ion diffusion. However, the performance improvement largely depends on the high operating voltage provided by the MnO_2_ cathode. Therefore, the device performance does not fully reflect the energy storage advantages of the MXene/carbon material composite itself.

**Figure 8:**
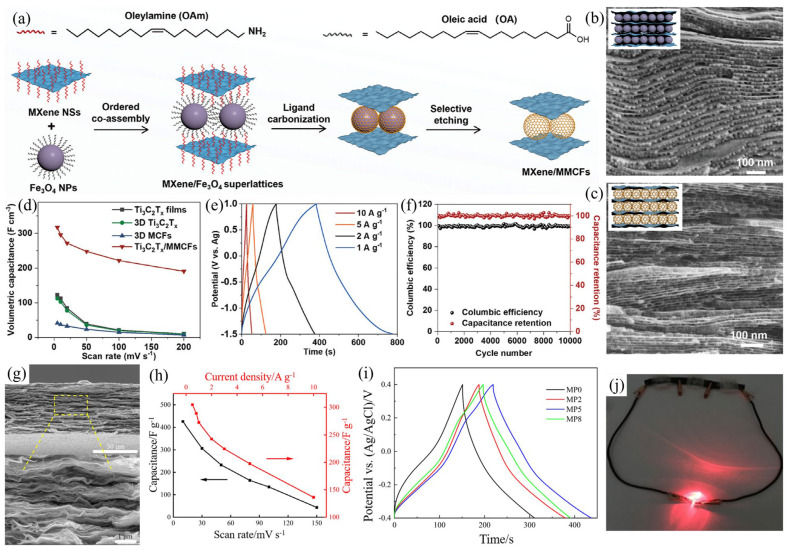
(**a**) Schematic diagram of MXene/MMCF composite synthesis process. Cross-section SEM images of (**b**) MXene/Fe_3_O_4_ superlattices and (**c**) MXene/MMCF superlattices. (**d**) Relationship curves between scan rates and the volumetric capacitance of the different MXene electrodes. (**e**) GCD curves of MXene/MMCFs at different current densities. (**f**) Cycle stability of the MXene/MMCFs at 10 A g^−1^. Reproduced with permission [119]. Copyright 2023 Wiley-VCH GmbH. (**g**) Cross-section SEM images of MXene/PANI film. (**h**) The specific capacitance of MXene/PANI under different conditions. (**i**) GCD curves of the different MXene/PANI film at 1 A g^−1^. (**j**) Optical photograph of a 1.8 V lamp illuminated by two MXene/PANI SSCs connected in series. Reproduced with permission [120]. Copyright 2022 Elsevier Ltd.

To further expand the applications of MXene in flexible substrates, Park et al. [118] prepared CNT/MXene@KTP conductive paper by alternately depositing Ti_3_C_2_T_x_ MXene and CNTs onto the surface of conventional fiber paper and then wove it into a spiral-shaped SSC. The device exhibited high volumetric capacitance and energy density, demonstrating the feasibility of using carbon materials and MXene in combination to construct flexible energy storage substrates. However, the energy storage performance of such conductive paper electrodes remains primarily limited by low active material loading. Their electrochemical performance remains to be further validated under conditions of high areal loading. Compared to conventional intercalation and composite strategies, Huang et al. [119] proposed a strategy for preparing alternately arranged MXene/single-layer mesoporous carbon framework (MMCF) superlattice structures (Figure 8a–c) via a self-assembly–carbonization–etching process. The ordered mesoporous carbon layers not only effectively suppress MXene self-stacking but also provide uniform ion diffusion channels and stable structural support. Therefore, MXene/MMCFs achieved a high volumetric capacitance of 317 F cm^−3^ and a wide voltage window of 2.5 V in a 1 M tetraethylammonium tetrafluoroborate/propylene carbonate (TEABF_4_/PC) organic electrolyte, with a capacitance retention rate approaching 100% during cycling (Figure 8d–f). This result indicates that constructing an ordered hierarchical structure can more effectively balance electron transport, ion diffusion, and volume utilization compared to simply increasing porosity or widening the interlayer spacing. However, such superlattice structures typically rely on complex multi-step synthesis processes, and their large-scale fabrication and cost control remain challenging.

Overall, the incorporation of carbon materials effectively mitigates MXene self-stacking and significantly enhances electrochemical performance by forming conductive networks and ion transport pathways. However, existing studies have not been comprehensive. Higher specific surface areas often come at the expense of volumetric energy density, while complex hierarchical structures increase both fabrication costs and process complexity. Therefore, the future development of MXene/carbon composite electrodes should focus on establishing structure–property relationships between structural parameters and energy storage. This approach will help harmonize the performance of flexible supercapacitors with practical application requirements.

### 4.3. MXene/Conducting Polymer Composite Electrodes

In the design of MXene composite electrodes, the incorporation of conductive polymers—such as polyaniline (PANI), polypyrrole (PPy), polythiophene (PTh) and its derivatives—is considered a key strategy for enhancing charge storage capacity [121,122,123]. In contrast to the carbon materials that mainly improve electron transport and structural stability, conductive polymers can provide additional pseudocapacitance through fast and reversible redox reactions, thus greatly enhancing the specific capacitance and energy density of the electrode. Moreover, the flexible polymer chains can intercalate into MXene layers, which can mitigate the re-stacking of nanosheets and maintain the ion transport channels, thus improving rate performance [122,123]. However, the inevitable volume expansion and contraction of conductive polymers during charging and discharging often lead to structural degradation and loss of activity. Consequently, balancing high pseudocapacitance contributions with long-term cycling stability remains a core challenge for these composite electrodes.

Li et al. [120] prepared flexible MXene/PANI (MP) composite films with adjustable interlayer spacing using a vacuum-assisted filtration method (Figure 8g). Adjusting the PANI content enabled precise control over the interlayer structure of MXene, thereby creating transport channels more conducive to ion diffusion. In a 1 M H_2_SO_4_ electrolyte, the MXene/PANI film exhibited a specific capacitance of 425.7 F g^−1^ (at 10 mV s^−1^), significantly outperforming the pure MXene electrode (Figure 8h,i). Furthermore, the flexible SSC assembled from MXene/PANI films achieved an energy density of 31.38 Wh kg^−1^ (1079.3 W kg^−1^) and successfully powered a 1.8 V lamp (Figure 8j). This result indicates that PANI not only serves as a pseudocapacitive active component but also acts as an interlayer scaffold to improve the ionic transport properties of MXene. However, the performance enhancement of this system largely depends on the redox contribution of PANI itself, which may face issues of conductive polymer structural fatigue during long-term cycling.

**Figure 9:**
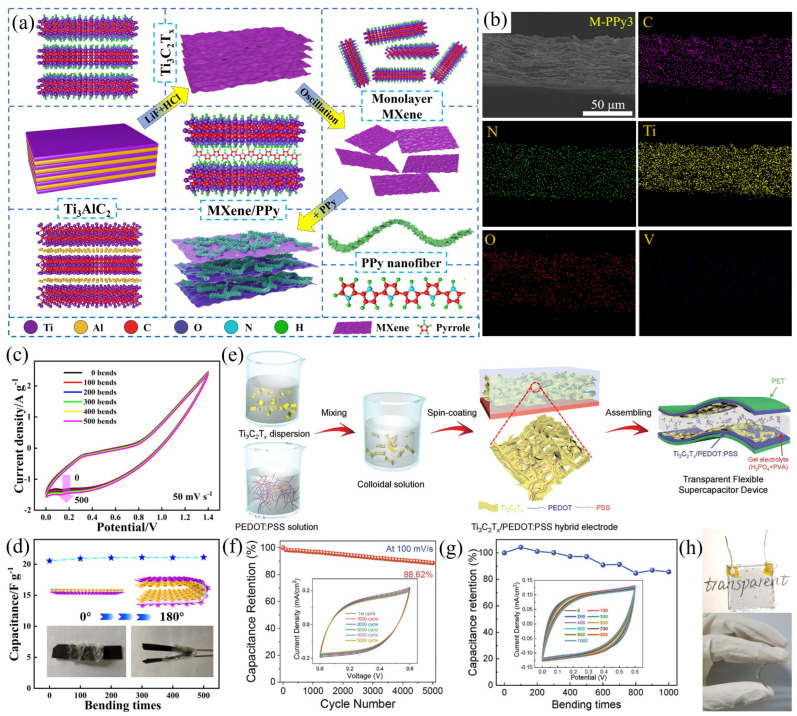
(**a**) Schematic diagram of MXene/PPy film synthesis process. (**b**) Cross-section SEM images and EDS element spectrums of MXene/PPy film. CV curves (**c**) and specific capacitance (**d**) of the M-PPy3//MnO_2_ ASC at different bending times. The illustration shows how the device bends. Reproduced with permission [36]. Copyright 2023 Elsevier Ltd. (**e**) Schematic diagram of the process for developing MXene/PEDOT:PSS SSC via blend spin-coating. Cycle stability (**f**) and mechanical flexibility test (**g**) of MXene/PEDOT:PSS SSC. (**h**) Optical images of the MXene/PEDOT:PSS SSC. Reproduced with permission [125]. Copyright 2024 Wiley-VCH GmbH.

To further enhance the energy storage capacity of flexible devices, Li et al. [36] replaced PANI with PPy nanofibers as the intercalation component (Figure 9a,b). With one-dimensional PPy nanofibers capable of forming a more open interlayer structure, the MXene/PPy electrode achieved a high specific capacitance of 563.8 F g^−1^ at 0.5 A g^−1^. Furthermore, it demonstrated excellent flexibility, with virtually no performance degradation after 500 bending cycles (Figure 9c,d). Meanwhile, the ASC constructed with an MXene/PPy anode achieved a high energy density of 35.3 Wh kg^−1^. However, its capacity retention after 6000 charge–discharge cycles was only 79.5%, indicating that although PPy can contribute to higher pseudocapacitance, the cycling stability is still limited by polymer volume changes. A similar phenomenon was observed in the study by Varghese et al. [124]. They prepared MXene-PANI and MXene-PPy composite electrodes via in situ polymerization. Their specific capacitances increased to 430 F g^−1^ and 305 F g^−1^, respectively, far exceeding that of pure MXene devices (105 F g^−1^). The SSC assembled from MXene-PANI achieved a high energy density of 38 Wh kg^−1^. These results further demonstrate that conductive polymers can effectively compensate for the insufficient pseudocapacitive contribution of MXene. However, existing studies have generally focused on improving initial capacitance and energy density. There has been insufficient attention paid to interfacial stability and polymer degradation mechanisms during long-term cycling. Therefore, the practical application potential of this system still requires further validation.

Apart from enhancing energy storage performance, conductive polymers also offer new avenues for the development of flexible and transparent energy storage devices. Ren et al. [125] utilized co-blend spin-coating to fabricate MXene/PEDOT:PSS transparent electrodes (Figure 9e). The MXene/PEDOT:PSS//MXene/PEDOT:PSS flexible SSC device prepared by this method not only exhibits excellent electrochemical stability (88.6%, 5000 cycles) and mechanical flexibility (86%, 1000 times) but also demonstrates overall good light transmittance (67.4%, 550 nm) (Figure 9f–h). Although its energy density is significantly lower than that of the PANI and PPy systems, this work demonstrates the potential for MXene-based SCs to be applied in transparent electronic devices and wearable systems.

Overall, the introduction of conductive polymers enhances the pseudocapacitive contribution and energy density of MXene electrodes and improves the ionic transport performance with the support of interlayers. However, the high specific capacitance achieved in most of the current work depends on the addition of high polymer content. Intrinsic problems of conductive polymers (such as volume expansion, structural aging, and interfacial delamination) have not been solved. Therefore, future research should focus on constructing stable confined structures or covalently linked networks to achieve a synergistic enhancement of high capacity, high stability, and excellent flexibility.

### 4.4. MXene/Transition Metal Compound Composite Electrodes

The introduction of transition metal compounds (TMCs)—including transition metal oxides (TMOs), layered double hydroxides (LDHs), and transition metal sulfides (TMSs)—has proven to be a key strategy for enhancing electrochemical activity and energy density during the development of MXene-based flexible SCs [126,127,128]. However, TMCs have inherently low electrical conductivity and are prone to structural degradation during charging and discharging. Consequently, when used alone, they often struggle to balance rate capability and cycling stability. In MXene/TMC composites, the presence of MXene enables the synergistic optimization of the conductive network and high-capacity active components.

Previously, Patra et al. [129] synthesized a MoWS_2_@BCN@MXene (MWSBX) ternary heterostructure via hydrothermal synthesis using BCN nanosheets and MXene nanosheets as growth templates. The SSC prepared by MWSBX achieved an areal capacitance of 289 mF cm^−2^ and a cycle retention rate of 91%. However, its energy density was only 19.73 μWh cm^−2^, indicating that while complex heterostructures facilitate interfacial synergy, they have not fundamentally overcome the energy storage limitations of flexible devices. Comparatively, the FeCo_2_O_4_@Fe Co_2_O_4_-MXene core–shell composite system developed by Chen et al. [130] demonstrated superior performance. The Fe Co_2_O_4_@FeCo_2_O_4_ nanospheres provide abundant redox active sites, while MXene forms a continuous conductive network and mitigates agglomeration of the active materials. Moreover, the composite electrode achieves a high specific capacitance of 1090.6 F g^−1^ at 1 A g^−1^ and maintains a capacity of 994 F g^−1^ at 10 A g^−1^, demonstrating excellent rate performance. The ASC assembled from FeCo_2_O_4_@FeCo_2_O_4_-MXen achieved an energy density of 49.8 Wh kg^−1^ and a capacity retention of 87.85% (after 20,000 cycles). This result indicates that constructing a heterojunction interface can effectively balance the relationship between conductivity and the number of active sites. Nonetheless, this system employs an activated carbon anode to form an asymmetric device, and its high energy density is partly attributed to the expanded voltage window of the device. This does not fully reflect the energy storage advantages inherent to the composite cathode itself.

**Figure 10:**
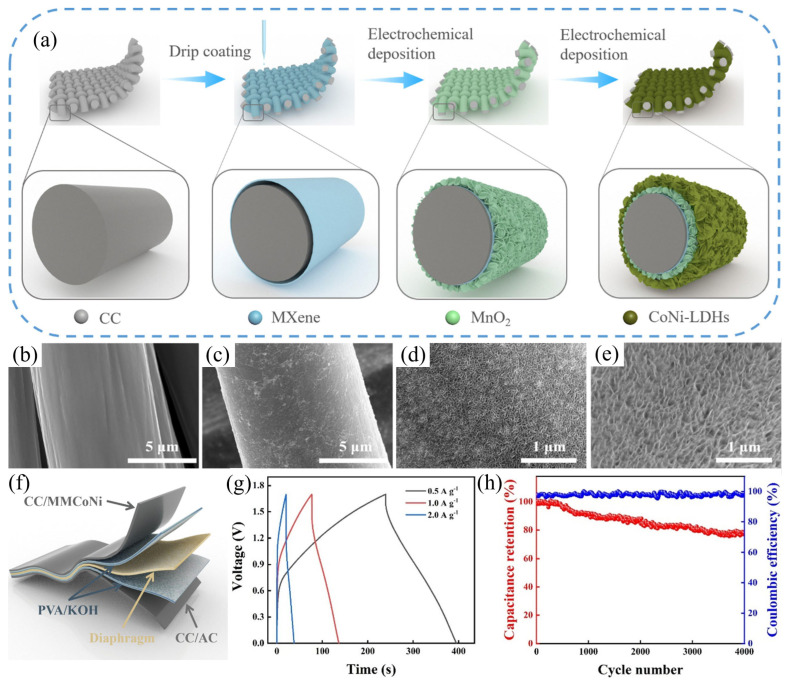
(**a**) Schematic diagram of CC/MMCoNi composite synthesis process. SEM images of (**b**) CC, (**c**) CC/MXene, (**d**) CC/MXene/MnO_2_, and (**e**) CC/MMCoNi. (**f**) Structural diagram of the CC/MMCoNi//AC ASC. (**g**) GCD curves of CC/MMCoNi//AC ASC at different current densities. (**h**) Cycle stability of CC/MMCoNi//AC ASC at 2 A g^−1^. Reproduced with permission [133]. Copyright 2022 Elsevier Ltd.

Apart from constructing heterostructures, regulating the interlayer structure of MXene through intercalation strategies is also a common approach. Luo et al. [131] prepared MXene/V_2_O_5_ composite films using vacuum-assisted filtration. The MV2 film electrode exhibited a specific capacitance of 319.1 F g^−1^ and a device energy density of 18.43 Wh kg^−1^. The introduction of V_2_O_5_ effectively increased the pseudocapacitive contribution while widening the interlayer spacing of MXene. However, the stability of the device was only 72.1% (after 8000 cycles), indicating that TMOs may still face structural degradation and interfacial instability issues during long-term cycling. Similar issues have also been observed in MoS_2_@MXene systems. Hayat et al. [132] developed a MoS_2_@MXene//Ti_3_C_2_ MXene ASC that exhibited excellent cycling stability (retaining 98% capacity after 10,000 cycles) and mechanical flexibility, but its energy density was only 1.21 Wh kg^−1^. These results indicate that while some TMSs can improve structural stability, they struggle to achieve high-capacity output at the same time. Unlike the aforementioned methods, as shown in Figure 10a–e, it has been reported that a hierarchical nano-arrayed CC/MXene-MnO_2_-CoNi-dihydroxide (CC/MMCoNi) composite material was successfully synthesized via multi-step electrodeposition on conductive carbon cloth (CC) [133]. When Co:Ni = 1:2, the CC/MMCoNi composite electrode exhibits a specific capacity of 922 F g^−1^ and achieves a high energy density of 65 Wh kg^−1^ with a wide voltage window of 1.7 V (Figure 10f–h). However, its capacity retention is only 79% (after 4000 cycles), indicating that as the number of active components increases, issues related to interfacial complexity and structural stability also intensify.

Overall, TMCs can significantly enhance the pseudocapacitive contribution and energy density of MXene composite electrodes, representing a key approach to breaking through the current performance limits of MXene energy storage. However, previous studies have primarily achieved high capacity by increasing the content of active components or constructing complex heterostructures. The trade-off is often longer ion diffusion paths, reduced structural stability, and more complex fabrication processes. Notably, the reported superior performance is often based on low surface loading and laboratory-scale testing, while studies on mass transfer behavior and long-term reliability under high-loading conditions remain relatively scarce. Therefore, future research should focus more on how MXene composites with transition metal compounds can achieve a balance between high energy density, high-rate performance, and a long life cycle.

TMCs typically possess abundant reversible redox sites that can provide significant pseudocapacitive contributions through Faradaic redox reactions, thereby compensating for the energy density limitations of MXene materials alone. Simultaneously, the high conductivity of MXenes effectively addresses the inherent low conductivity of TMCs, facilitating rapid electron transport and reducing interfacial resistance. Additionally, 2D MXene sheets can serve as growth substrates or conductive frameworks to regulate the nanostructural distribution of TMCs, preventing their agglomeration or restricting bulk diffusion, thereby enhancing active site utilization. Through interfacial engineering and heterostructure construction, MXene/TMC composite systems achieve synergistic optimization of conductive networks and high-capacity active components, demonstrating significant advantages in the development of high-performance flexible SCs.

## 5. Key Challenges and Mitigation Strategies

**Figure 11:**
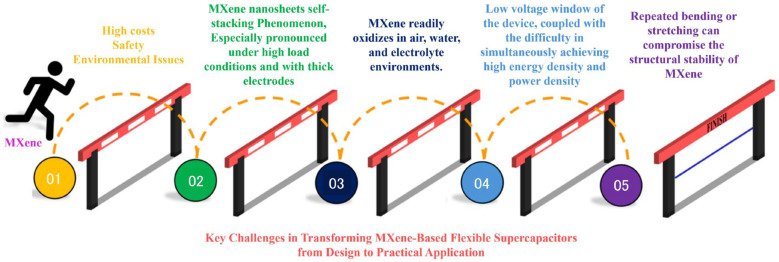
Key challenges in transitioning MXene-based flexible supercapacitors from design to practical application.

Despite the impressive performance of MXene and its composites in flexible SCs, numerous challenges remain in advancing their application from structural design to practical implementation (Figure 11). This section will focus on the key challenges encountered when utilizing MXene and its composites in flexible SCs, along with corresponding mitigation strategies.

(1) Affected by intermolecular forces, MXene nanosheets exhibit self-stacking behavior during operation, particularly pronounced under high loading and thick electrode conditions. To address this issue, introducing carbon materials, conductive polymers, or TMCs as interlayer spacers can effectively mitigate MXene self-stacking. Additionally, constructing heterojunctions or three-dimensional porous structures can also effectively expand ion transport pathways [102,109].

(2) The abundance of surface functional groups results in insufficient oxidation stability of MXene during long-term operation. To address this issue, terminal group capping of MXene using reagents such as sodium citrate can reduce the likelihood of surface functional groups reacting with air, water, and electrolyte environments [134]. Additionally, surface coating, the construction of carbon/polymer protective layers, and the device encapsulation design can also enhance the stability of MXene-based SCs to a certain extent [135].

(3) The voltage window and energy density of single MXene devices are relatively limited, failing to meet practical application requirements. To address this issue, a common strategy involves compositing MXene with conducting polymers or TMCs to enhance energy density through multi-electron redox reactions. Additionally, constructing asymmetric or hybrid SCs to broaden the device’s operating voltage window serves as a convenient approach to achieve synergistic improvements in both energy and power density.

(4) Repeated bending or stretching can compromise the structural stability of MXene. To address this issue, interlayer interactions can be enhanced by introducing flexible scaffold materials. Additionally, designing pleated or fibrillated structures and fabricating coaxial fiber electrodes are effective approaches to improving the mechanical adaptability of electrodes.

(5) Beyond the aforementioned challenges, high costs, safety concerns, and environmental issues also constrain MXene development. The difficulty in synthesizing high-quality MAX phase precursors leads to high costs for batch production of MXene. Traditional HF or fluoride salt etching methods generate toxic waste, posing safety hazards and environmental pollution. Currently, research is exploring free-F synthesis methods, such as molten salt etching, electrochemical etching, and chemical vapor deposition, for preparing high-performance MXene materials. However, these methods remain immature and yield low production rates.

Therefore, in addition to addressing issues such as MXene nanosheets’ self-stacking, insufficient oxidation stability, limited energy density, and structural degradation under complex deformation conditions, there remains a need to develop low-cost, safe, efficient, and scalable MXene preparation methods.

## 6. Conclusions and Prospectives

Overall, MXene and its composites have emerged as a significant material system in the research of flexible SC electrodes. Table 4 and Table 5 summarize and compare the storage capabilities of selected MXene materials and composites, along with their applications in flexible SCs. However, issues such as sheet self-stacking, insufficient oxidation stability, limited energy density, and structural degradation under complex deformation conditions constrain further performance enhancement. By optimizing synthesis methods and constructing composite systems with carbon materials, conducting polymers, and transition metal compounds, it is possible to effectively expand ion transport pathways, enhance electrochemical activity, and improve mechanical stability, thereby achieving simultaneous improvements in energy density, power density, and flexibility. Future research should not be confined to optimizing individual parameters. Instead, it should pursue a balanced approach through structural design, interfacial engineering, and coordinated device-level regulation to achieve equilibrium among energy density, power density, stability, and manufacturability. This will advance the practical application of these materials in flexible energy storage devices.

## Figures and Tables

**Table 1:** Different HF concentrations on the structure and electrochemical performance of Ti_3_C_2_T_x_ MXene.

HF Concentrations	Etching Condition	Structural Features	Electrochemical Performance	Ref.
5 wt.%	50 °C, 24 h	Multilayer structure is intact with few defects; Al-layer etching is incomplete	Excellent electrical conductivity but poor charge storage capacity	[71]
10 wt.%	25–35 °C, 24 h	Multilayer structure is intact with few defects; outstanding oxidation resistance	Excellent cycling stability; rate performance needs improvement	[70]
20–30 wt.%	25–40 °C, 18–36 h	Complete etching of the Al layer; increased interlayer spacing; suitable level of defects and terminal groups	Excellent electrical conductivity, rate capability, and charge storage capacity; cycling stability needs improvement	[72]
40 wt.%	25–40 °C, 24–30 h	Etching is complete; multilayer structure is clearly visible; Ti vacancies and corrosion are present at the edges	Great magnification performance and high charge storage capacity; low conductivity and stability	[69]
48 wt.%	25 °C, 24 h	Multilayer structure is evident; increased number of -F terminal functional groups	Enhanced capacitive activity; high charge transfer impedance; insufficient rate capability and cycling stability	[70]

**Table 2:** Advantages and limitations of different fluoride salt etching systems for MXene synthesis.

Fluoride Salt System	Advantages	Limitations	Ref.
LiF/HCl	High stripping efficiency; MXene nanosheet integrity	Long etching time; high content of -F groups	[62]
NaF/HCl	Low cost	Low stripping efficiency	[74]
FeF_3_/HCl	Promotes interlayer spacing expansion	Residual Fe^3+^; local impurity phases	[75]
NH_4_F or NH_4_HF_2_	Mild etching process with high safety; uniform interlayer structure; large specific surface area	Slow etching rate and low stripping efficiency; abundant -F groups	[73,76]

**Table 3:** Advantages and limitations of different electrochemical etching systems for MXene synthesis.

Electrolyte System	Advantages	Limitations	Ref.
HBF_4_	BF_4_^−^ promotes the dissolution of A-layer atoms; large-sized nanolayers with few defects	Some -F functional groups remain; low etching rate	[89]
NaBF_4_/HCl	Improved etching rate; H_2_ bubble-assisted stripping; continuous etching	Some -F functional groups remain; complex process parameters	[90]
Cl^−^-based electrolytes	Free-F functional groups; environmentally friendly; enhances conductivity by introducing -Cl functional groups	Low etching rate; technology is not mature; scope of application is limited	[67]

**Table 4:** Comparative electrochemical performance of different electrode materials in SC applications.

Method	Electrode	Electrolyte	Capacitance	Condition	Cycles	Ref.
Freeze-drying	MXene film-2	1 M H_2_SO_4_	296 F g^−1^	2 mV s^−1^	—	[110]
Vacuum filtration, freeze-drying	Zn-A-MXene film	1 M H_2_SO_4_	465.1 F g^−1^	1 A g^−1^	—	[111]
Bidirectional freeze-casting, freeze-drying	A-MHA-40%	1 M H_2_SO_4_	760 F g^−1^	1 A g^−1^	10,000, 97%	[112]
Vacuum filtration	MXene/rGO/CNTs film	1 M H_2_SO_4_	463.5 F g^−1^	1 A g^−1^	—	[82]
Self-assembly, carbonization etching	MXene/MMCFs	1 M TEABF_4_/PC	157 F g^−1^	1 A g^−1^	10,000, 100%	[119]
Hydrothermal method, vacuum filtration	MXene/PANI film	1 M H_2_SO_4_	425.7 F g^−1^	10 mV s^−1^	—	[120]
Sacrificial template, vacuum filtration	MXene/PPy film	1 M H_2_SO_4_	563.8 F g^−1^	0.5 A g^−1^	6000, 79.5%	[36]
Dissolution heat, self-assembly	FeCo_2_O_4_@FeCo_2_S_4_-MXene	1 M KOH	1090.6 F g^−1^	1 A g^−1^	5000, 91.47%	[130]
Vacuum filtration	MXene/V_2_O_5_ film	1 M H_2_SO_4_	319.1 F g^−1^	0.5 A g^−1^	5000, 70.4%	[131]
Multi-step electrodeposition	CC/MXene-MnO_2_-CoNi	1 M KOH	922 F g^−1^	1 A g^−1^	—	[133]

**Table 5:** Summary of MXene-based flexible SCs.

Device	Electrolyte	Energy Density	Power Density	Flexibility	Cycles	Ref.
Zn-A-MXene//Zn-A-MXene	1 M PVA/H_2_SO_4_	9.55 Wh kg^−1^	603.16 W kg^−1^	180°, 100%	5000, 81.25%	[111]
A-MHA-40%//CNT sponge	1 M H_2_SO_4_	3.4 Wh kg^−1^	100 W kg^−1^	—	10,000, 98%	[112]
CNT/MXene7@KTP//CNT/MXene7@KTP	1 M PVA/H_2_SO_4_	2.06 mWh cm^−3^	73.94 mW cm^−3^	135°, 95%	5000, 95.5%	[118]
TSC3 aerogel//TSC3 aerogel	1 M PVA/H_2_SO_4_	35.12 mWh cm^−2^	0.039 mW cm^−2^	180°, 10,000 cycles, 90.51%	10,000, 91.23%	[117]
MXene/rGO/CNTs//MnO_2_	1 M PVA/H_2_SO_4_	33.95 Wh kg^−1^	814.8 W kg^−1^	180°, 500 times, 101.6%	8000, 92.9%	[82]
MP5//MP5	1 M PVA/H_2_SO_4_	31.18 Wh kg^−1^	1079.3 W kg^−1^	—	4000, 71.4%	[120]
M-PPy3//MnO_2_	1 M PVA/H_2_SO_4_	35.3 Wh kg^−1^	486.1 W kg^−1^	180°, 500 times, 100%	6000, 86.8%	[36]
MXene-PANI//MXene-PANI	1 M H_2_SO_4_	38 Wh kg^−1^	800 W kg^−1^	—	10,000, 84%	[124]
MXene/PEDOT:PSS//MXene/PEDOT:PSS	PVA/H_3_PO_4_	0.07 μWh cm^−2^	42 μW cm^−2^	bending radius of 1.0 mm, 1000 times, 86%	5000, 88.6%	[125]
MWSBX//MWSBX	0.5 M PVA/K_2_SO_4_	19.73 μWh cm^−2^	538.09 μW cm^−2^	—	5000, 91%	[129]
FeCo_2_O_4_@FeCo_2_S_4_-MXene//AC	1 M PVA/KOH	49.8 Wh kg^−1^	800 W kg^−1^	—	20,000, 87.85%	[130]
MXene/V_2_O_5_//MXene/V_2_O_5_	1 M PVA/H_2_SO_4_	18.43 Wh kg^−1^	603.2 W kg^−1^	180°, 500 times, ~110%	8000, 72.1%	[131]
MXene/V_2_O_5_//MnO_2_	1 M PVA/H_2_SO_4_	20.83 Wh kg^−1^	374.94 W kg^−1^	180°, 500 times, 100%	8000, 83.9%	[131]
MoS_2_@MXene//MXene	2 M KOH	1.21 Wh kg^−1^	54.45 W kg^−1^	90°, 100 times, ~95%	10,000, 98%	[132]
CC/MXene-MnO_2_-CoNi//AC	1 M PVA/KOH	65 Wh kg^−1^	1491 W kg^−1^	90°, 500 times, 100%	4000, 79%	[133]

## Data Availability

No new data were created or analyzed in this study. Data sharing is not applicable to this article.
